# Parafoveal and Peripapillary Perfusion Predict Visual Field Recovery in Chiasmal Compression due to Pituitary Tumors

**DOI:** 10.3390/jcm9030697

**Published:** 2020-03-04

**Authors:** Ga-In Lee, Kyung-Ah Park, Sei Yeul Oh, Doo-Sik Kong

**Affiliations:** 1 Department of Ophthalmology, Samsung Medical Center, Sungkyunkwan University School of Medicine, Seoul 06351, Korea; porishi71@gmail.com (G.-I.L.); kparkoph@skku.edu (K.-A.P.); 2 Department of Neurosurgery, Endoscopic Skull Base Surgery Clinic, Brain Tumor Center, Samsung Medical Center, Sungkyunkwan University School of Medicine, Seoul 06351, Korea

**Keywords:** chiasmal compression, visual field recovery, prognosis, optical coherence tomography angiography

## Abstract

Background: To evaluate the potential of vessel density alterations for predicting postoperative visual field (VF) improvement in chiasmal compression using optical coherence tomography angiography (OCT-A). Methods: The study investigated 57 eyes of 57 patients diagnosed with pituitary tumors and 42 eyes of 42 age and refractive error matched controls. All eyes with chiasmal compression for which preoperative optical coherence tomography (OCT) and OCT-A, and pre- and postoperative VF data were available. Preoperative vessel densities of superficial retinal capillary plexus (SRCP), deep retinal capillary plexus (DRCP), and radial peripapillary capillary (RPC) segment were utilized by OCT-A. Results: Preoperative peripapillary retinal nerve fiber layer and ganglion cell layer complex thickness and vessel densities of SRCP and RPC segments in eyes with chiasmal compression were significantly reduced compared with healthy controls (*p* < 0.001, *p* < 0.001, *p* = 0.007, and *p* = 0.020, respectively). In multivariate regression analysis, preoperative perimetric mean deviation (MD) (*p* = 0.002) and vessel density of SRCP (*p* = 0.025) were correlated significantly with postoperative perimetric MD. Spearman’s correlation analysis revealed significant correlations between preoperative MD on perimetry (r = 0.443, *p* = 0.001), vessel densities of SRCP (r = 0.288, *p* = 0.035) and RPC segment (r = 0.347, *p* = 0.009), and postoperative perimetric MD. Conclusions: Structural degeneration referred to as microvascular alterations measured by OCT-A and preoperative VF defects were associated with worse postoperative VF prognosis. Parafoveal and peripapillary vessel densities may serve as a sensitive, structural prognostic factors in the preoperative judgement of chiasmal compression.

## 1. Introduction

A growing pituitary tumor that compresses optic chiasm can increase the risk of injury to the retinal ganglion cell axons, resulting in deterioration of visual function including visual field (VF) and visual acuity. Previous studies reported that the degree of visual function recovery depends on the degree of initial damage to retinal ganglion cell axons [1,2]. Optical coherence tomography (OCT) has been widely used to assess the morphological and pathological alterations in compressive optic neuropathy. Preoperative retinal nerve fiber layer (RNFL) thickness has been correlated strongly with postoperative VF defects in various studies on chiasmal compression [3,4,5]. Also, the thickness of retinal layers [6,7] and ganglion cell layer complex (GCC) thickness [8,9] or area [3] have been used to predict visual function after decompression surgery in patients with chiasmal tumors. Recent advances in OCT-angiography (OCT-A) to reconstruct the three-dimensional vascular structures of the retinal layers and peripapillary areas with auto-segmentation, have led to the identification of microvascular alterations in various optic neuropathies such as glaucoma [10], ischemic optic neuropathy [11] or inflammatory optic neuropathy [12]. Vessel density was significantly correlated with the severity of VF loss in patients with glaucoma [10]. However, to date, no study has evaluated the usefulness of vessel density measurement using OCT-A as a predictor of postoperative VF recovery in chiasmal lesions. In this study, we evaluated the potential role of OCT-A in the measurement of parafoveal and peripapillary vessel densities to predict postoperative visual recovery in chiasmal compression due to pituitary tumors.

## 2. Materials and Methods

This retrospective cohort study has been approved by the Institutional Review Board of Samsung Medical Center (Seoul, Republic of Korea) (IRB No. 2018-07-144, Approved date: 1 August 2018). The data collection followed the tenets of the Declaration of Helsinki. This study included patients with chiasmal compression caused by brain tumors and healthy controls who visited the Department of Neuro-ophthalmology and Neurosurgery of Samsung Medical Center from 1 August 2018, to 31 June 2019. The study involved patients with evidence of optic neuropathy or chiasmal syndrome based on compatible VF defects or decreased visual acuity and chiasmal compressive tumors confirmed by magnetic resonance imaging. All patients underwent trans-sphenoidal tumor resections. Patients visited the clinic postoperatively at 4–6 months for ophthalmic examinations. Only patients who were followed up for more than 3 months after tumor resection were included in the analysis. Only a single eye (the one with the worse VF defects) based on mean deviation (MD) of each patient was selected for the analysis. Patients and healthy controls with other ophthalmic diseases (glaucoma, a refractive error greater than 6.0 diopters of spherical equivalent as high myopia and hyperopia or 3.0 diopters of astigmatism in either eye, amblyopia, epiretinal membrane, age-related macular degeneration, diabetic retinopathy, retinal artery/vein occlusion, optic neuritis, and other ischemic optic neuropathy), and previous retinal surgery, which may affect vessel density, and those with known systemic or inflammatory diseases such as cancer, and multiple sclerosis were excluded. Healthy controls were recruited from staff and healthy volunteers who visited the hospital for routine eye examination. Written informed consent was obtained. None of the controls had a history of ocular or neurologic disease. Healthy controls were required to carry normal optic discs, normal thickness of intra-retinal layers, and intraocular pressure (IOP) < 21 mm. All eyes were evaluated with fundus photography and Cirrus High-Definition (HD)-OCT (Carl Zeiss Meditec AG, Jena, Germany). VF perimetry for patients with chiasmal compression was performed with a Humphrey Field Analyzer using the 30-2 SITA-standard protocol (Humphrey 740 Visual Field Analyzer, Carl Zeiss Meditec Inc. Dublin, CA, U.S.A). Only reliable VFs (≤ 33% false positives or false negatives, and fixation losses < 20%) have been considered. The MD was used for the analysis.

All patients and healthy controls underwent Cirrus HD-OCT. The pRNFL thickness was measured using the optic disc cube 200 × 200 protocol with Cirrus software. This protocol generated a cube of data via a 6-mm-square grid. A circle with a 3.46 mm diameter was automatically centered on the optic disc. This analytical protocol yields an average RNFL thickness, maps four quadrants (superior, inferior, nasal, and temporal), and classifies results compared with an internal normative database. Only scans with a signal strength ≥ 6 without motion artifacts have been included. For the GCC analysis, the thickness of the ganglion cell layer and the inner plexiform layers (IPL) were evaluated. The GCC thickness was automatically measured at various locations around the fovea (superior, temporal, nasal, and inferior). The thickness of each of the four quadrants was calculated.

The retinal and peripapillary microvasculature was analyzed using a Topcon OCT instrument (DRI OCT Triton Plus) in all patients and healthy controls. All OCT-A imaging was performed 3 to 6 months after tumor resection. The Triton swept-source OCT uses a wavelength of 1050 nm with a scan speed of 100,000 A-scans per second. For each field scan, three repeated B-scans obtained from 500 uniformly spaced locations were sequentially acquired in order to verify the repeatability of vessel density measurement. Each B-scan consisted of 500 A-scans and the inter-scan time between repeated B-scans was about 5 ms, accounting for the mirror scan duty cycle. The instrument uses an active eye tracker which follows fixation movement. It detects blinking and adjusts the scan position accordingly, thereby reducing motion artifacts during OCT-A image generation. Each patient underwent two imaging sessions consisting of a peripapillary scan (4.5 × 4.5 mm diameter) centered on the optic disc and a 3.0 × 3.0 mm diameter perifoveal scan centered on the macula. The superficial and deep retinal capillary plexuses were separated automatically via layer segmentation with the OCT instrument software (IMAGEnet 6 V.1.14.8538). The OCT performs automated segmentation and offers several preset reference boundaries for en- face projection, with a final rendered depth scale of 2.6 µm/voxel. Swept source OCT-A evaluation included vessel density, foveal avascular zone area, presence of vascular abnormalities such as dilated endings of the capillaries and a number of choriocapillaris flow voids. The superficial retinal capillary plexus (SRCP) extended from 3 µm below the internal limiting membrane (ILM) to 15 µm below the IPL, while the deep retinal capillary plexus (DRCP) extended from 15 to 70 µm below the IPL, according to a previously validated method by Park et al. [13]. The radial peripapillary capillary (RPC) segment extended from the ILM to the posterior boundary of the RNFL. Vessel density was defined as the percentage of the area occupied by vessels in a localized region. The software automatically fitted an Early Treatment Diabetic Retinopathy Study (ETDRS) grid pattern circular to the optic disc and generated vessel density for each layer with high repeatability and reproducibility [14,15]. The pericentral vessel density was defined as the mean of the four quadrants in the ETDRS grid except the central circular area. Five areas (center, nasal, temporal, superior, and inferior) dividing the center of the macula and disc are displayed. The blood vessel density of each area is indicated as a percentage. All participants underwent both OCT-A and Cirrus HD-OCT imaging on the same day. We excluded eyes with low image quality < 40 and those showing a partial decrease in image intensity. Trained graders reviewed all images and excluded eyes with large eye movements during image capture reflected in motion artifacts involving more than three lines and any discontinuities of blood vessels in the OCT-A images. The location of the optic disc margin was reviewed for accuracy and the margin was adjusted manually as needed and confirmed by the graders (G.I.L., K.A.P.).

The data are presented as the mean ± standard deviation (SD). The best corrected visual acuity (BCVA) was converted to a Logarithm of the Minimum Angle of Resolution (log MAR) scale. Student’s t-test and Wilcoxon rank sum test were used to compare age, spherical equivalent (SE), and preoperative MD of VF between patients with chiasmal compression preoperatively and healthy controls. The normal distribution of continuous variables was confirmed by the Kolmogorov-Smirnov test. Student’s t-test and Wilcoxon rank-sum test were decided based on the normality test. In the normality test, the Wilcoxon rank-sum test was used with a *p* value < 0.05 and t-test for *p* value > 0.05. Linear regression analysis was conducted after adjusting for age and SE to compare vessel densities between patient and healthy controls. Univariate and multivariate (backward selection) linear regression analysis adjusting for age and SE were performed to determine predictive factors for VF recovery after decompression surgery. Only the parameters with a *p* value less than 0.1 in the univariate analysis were included in the multivariate analysis. Spearman’s correlation coefficients were calculated to analyze the correlation between microvascular densities and intra-retinal layer thicknesses and postoperative VF MD. A *p* value less than 0.05 was considered statistically significant. All statistical analyses were performed with R 3.5.1 (Vienna, Austria; http://www.R-project.org/).

## 3. Results

The study included 57 eyes of 57 patients with chiasmal compression and 42 eyes of healthy controls. These patients showed chiasmal compression due to pituitary adenoma (43, 75.4%), meningioma (7, 12.3%), craniopharyngioma (3, 5.3%), and Rathke’s cleft cyst (4, 7.0%). The mean age of patients and healthy controls was 49 ± 13 (range, 20–66) and 49 ± 11 (range, 22–72) years, respectively. The mean duration of the visual symptoms was 5 ± 11 months and ranged from 1 to 24 months. There were no statistical differences in age, gender, or SE between the two groups (Table 1). Preoperative VF MD varied significantly between the two groups (*p* < 0.001). No disc swelling was detected in the patient group upon fundus examination or in the color-coded maps of pRNFL thickness of OCT. Both the pRNFL and GCC were significantly thinner in the patient group than in the control group (all *p* < 0.001) preoperatively. Preoperative vessel densities of SRCP and RPC segments were significantly decreased compared with those of healthy controls (Table 2).

Results from the univariate and multivariate linear regression analyses for VF MD as a dependent variable are summarized in Table 3 and Table 4. Results of univariate analysis showed that preoperative VF MD (*p* = 0.002), vessel densities of SRCP (average, *p* = 0.017; superior, *p* = 0.049) and RPC segments (average, *p* = 0.003; superior, *p* = 0.032) were associated with postoperative VF results (Table 3).

When only average values of OCT-A were included in the multivariate analysis, the preoperative VF MD (*p* = 0.002) and the average vessel density of SRCP (*p* = 0.025) were significantly associated with postoperative VF outcome. Multivariate regression analysis using superior sector vessel densities instead of average value of SRCP and RPC segments showed that preoperative VF MD (*p* = 0.001) and the average vessel density of SRCP (*p* = 0.013) were significantly associated with postoperative VF outcome (Table 4).

**Figure 1:**
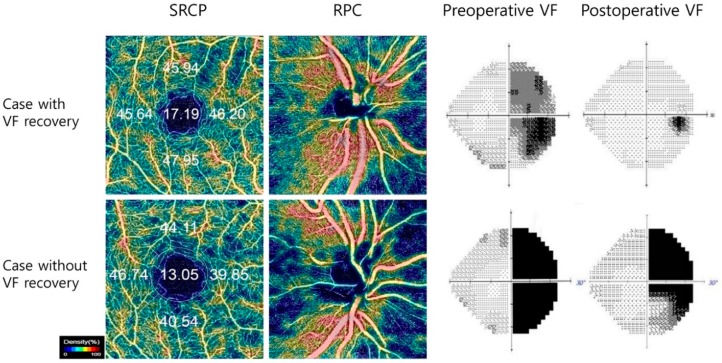
Two representative cases based on the degree of visual field (VF) recovery diagnosed with pituitary adenoma who underwent transsphenoidal tumor resection. In preoperative stage, OCT-A color-coded density maps showed in the superficial retinal capillary plexus (SRCP) and radial peripapillary capillary (RPC) segment. Preoperative vessel densities in the SRCP and RPC segment were correlated with postoperative VF recovery.

**Figure 2:**
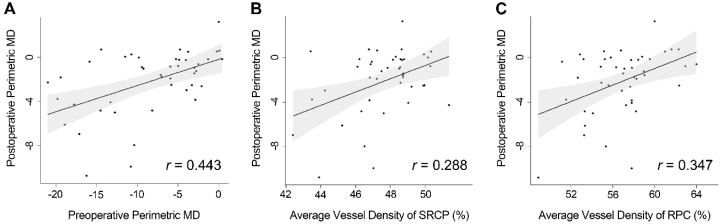
Scatterplots illustrate the correlation between postoperative mean deviation (MD) of visual field (VF) and preoperative MD of VF (r = 0.443, *p* = 0.001) **(A)**, vessel densities of superficial retinal capillary plexus (SRCP) (r = 0.288, *p* = 0.035) **(B),** radial peripapillary capillary (RPC) segment (r = 0.347, *p* = 0.009) **(C)**. The black line indicates the linear regression line and the dark gray area the corresponding 95% CI.

The degree of correlation between preoperative perimetric MD and OCT-A measures of SRCP and RPC segments with postoperative perimetric MD is presented in Figure 1 and Figure 2. The correlation between preoperative perimetric MD (r = 0.443, *p* = 0.001) and postoperative perimetric MD, and the association between vessel densities of SRCP (r = 0.288, *p* = 0.035) and RPC (r = 0.347, *p* = 0.009) and postoperative perimetric MD were all significant in Spearman correlation analysis.

## 4. Discussion

After the recent advent of OCT-A facilitating the reconstruction of the three-dimensional vascular structure of the retinal layers and peripapillary areas, its parameters were actively studied in various types of optic neuropathy [10,11,12,16]. OCT-A parameters reflect anatomical changes around optic nerve head and predict VF progression in other types of optic neuropathy [10,11,12,16]. However, few studies reported the role of OCT-A in chiasmal compression [17]. Our study evaluated the prognostic value of preoperative OCT-A parameters in chiasmal compression, for the first time. The results showed significant correlation between postoperative VF recovery and preoperative perimetric MD and superficial parafoveal microvasculature on OCT-A. The findings suggested that OCT-A parameters serve as sensitive, structural prognostic factors in chiasmal compression.

Parafoveal and peripapillary vessel densities are closely associated with the severity of VF damage in advanced open angle glaucoma [16]. Yarmohammadi et al. reported that vessel density on OCT-A showed a stronger correlation with VF than RNFL thickness, and that the role of reduced vessel density was independent of structural changes such as RNFL thinning in patients with primary open angle glaucoma [10]. However, few studies demonstrated a decrease in retinal perfusion using OCT-A in eyes with chiasmal compression [17]. Previously, Higashiyama et al. analyzed retinal vessel density using OCT-A in four patients with chiasmal lesions and reported that retinal perfusion defects in the corresponding area of injury correlated with VF defects [17]. In this study, we also found that significant differences in preoperative retinal and peripapillary vessel density in eyes with chiasmal compression compared with healthy controls. Our preoperative results are consistent with the study reported by Higashiyama et al. [17]. The decreased perfusion in these patients may be attributed to a reduced metabolic demand due to optic atrophy [18,19,20]. According to one hypothesis, the neuronal and axonal decline reduces the metabolic activity within the inner retinal layers, subsequently lowering the oxygen and blood demand and resulting in regression of the superficial vessels [20]. The deep retinal plexus vascularized by the anastomoses of superficial vessels may be secondarily affected [20]. In our previous cross-sectional study, we found significant correlations between peripapillary vessel density of RPC segment and pRNFL thickness in chiasmal compression [21]. These findings suggest pathophysiological mechanisms leading to intraocular microvascular changes secondary to optic atrophy.

However, in this study, while the intraocular vessel density has a significant predictive value for the postoperative visual outcome, the pRNFL thickness, which represents the degree of axonal damage, did not show a significant predictive value. We postulate that although the reduction of vessel density might occur secondary to axonal damage, the intra-retinal vascular changes might be amplified resulting in increased sensitivity of detection compared with pRNFL thickness. We suggest that the discrepancy in the predictive power of OCT parameters compared with OCT-A parameters might be due to the difference in the innate sensitivity of the measurements, and not because of the temporal sequence of the injury.

Preoperative visual field changes in chiasmal compression are powerful predictors of postoperative visual function [6,7,22,23,24]. According to a study by Gnanalingham et al., involving a total of 41 patients, 95% of patients recovered from VF defects after decompression surgery, and the degree of recovery was correlated with the degree of preoperative VF defects [22]. Since then, several studies reported preoperative VF defects as independent predictors of postoperative visual recovery [6,7,23,24]. In this study, preoperative VF MD were also potent factors predicting postoperative VF recovery, consistent with prior studies [6,7,22,23,24].

According to the first published OCT study on chiasmal compression by Danesh-Meyer et al., the RNFL thickness showed a strong correlation with VF defects, with a higher number of significant changes in the horizontal than in the vertical sector, and a stronger correlation in the temporal than in the nasal sector [1]. The study showed that OCT measurements of the RNFL accurately reflect the degree of VF loss with distinct patterns of ganglion cell loss [1]. In chiasmal compression, the axons that are closest to the expanding mass are those crossing from the nasal hemiretinal ganglion cells primarily. The temporal hemiretinal fibers are involved in an enlarged tumor and affect the non-crossing fibers. These pathophysiological changes due to tumor compression may contribute to varying patterns of injury to ganglion cell axons based on location. Subsequent studies reported that RNFL thickness and GCC area or thickness influence visual outcomes following decompression surgery [3,5]. Jacob et al. reported limited VF recovery in eyes with RNFL thinning independent of age and duration of symptoms [5]. In their study, the inferior sector of RNFL thickness showed a robust prognostic correlation with VF outcome after 3 months postoperatively [5]. When the RNFL thickness increased by 1 micron, the probability of complete VF recovery increased 6.31-fold [5]. Moon et al. also reported that the preoperative RNFL thickness in the temporal sector and GCC area correlated significantly with postoperative VF recovery [3]. Subsequent studies also reported that RNFL and GCC thickness play an important role in predicting postoperative visual outcome [4,9,23,25,26].

In this study, in contrast to previous studies, no statistically significant correlation existed between preoperative RNFL thickness and GCC thickness and postoperative VF recovery. In univariate analysis, only GCC thickness showed a marginally significant correlation with postoperative VF recovery, but this was not significant in multivariate analysis. The differences in study results for pRNFL and GCC may be attributed to differences in the characteristics of patients included in each study such as patients’ age, disease duration, and severity of chiasmal compression. Previous studies may have included higher numbers of patients with severe preoperative RNFL thinning compared with our study. In our study, the average preoperative pRNFL was 91 μm, which was thicker than the average in previous reports (range, 73–88 μm) [1,3,8,27,28]. The average preoperative MD on the perimetry was also −7.27 dB in our study, higher than in other studies (range, −9.62 to −17.5 dB) [1,3,6,7,29]. Therefore, this study may have included a higher number of cases with mild VF MD and fewer structural changes, which may have resulted in a lower statistical significance in the correlation analysis of pRNFL and GCC thickness.

There are several possible limitations in this study. First, our data was followed for 3 to 6 months after decompression surgery, which was a relatively short-term period. Further studies are needed to correlate long-term visual outcomes and predictive factors. Second, the vessel density measurement using OCT-A which the angiographic signal is based on movement, but there are many other factors that comprise measurement of perfusion. Further, we could not rule out the possibility of undetected micro-swelling of the optic disc/mild axonal edema affecting the evaluation of altered vessel density.

However, this study for the first time demonstrated the prognostic value of vessel density using OCT-A in patients with chiasmal compression treated with tumor resection. Measurement of vessel densities by OCT-A will help elucidating the pathophysiology of chiasmal compression and provide a valuable tool to predict postoperative outcomes of visual function.

## Figures and Tables

**Table 1:** Baseline characteristics of patients with chiasmal compression and healthy controls.

	Patients (*n* = 57)	Healthy Controls (*n* = 42)	*p* value
Age	49 ± 13	49 ± 11	0.749 *
Gender (M/F)	22/35	17/25	1.000 ^†^
Spherical equivalent	−1.14 ± 1.98	−1.32 ± 1.84	0.316 ^‡^
Symptom duration (mon)	5 ± 11	-	
Preoperative VF (MD)	−7.27 ± 6.20	0.07 ± 1.18	<0.001 ^‡^

*n* = numbers; M = male; F = female; VF = visual field; MD = mean deviation. * *p* value by Student’s t-test. ^†^ *p* value by Chi-squared test. ^‡^ *p* value by Wilcoxon rank sum test. * T-test and ^‡^ Wilcoxon rank sum test was decided by normality test. In the normality test, Wilcoxon rank sum test was used for a *p* value < 0.05 and t-test for *p* value > 0.05.

**Table 2:** Comparison of intraretinal layer thickness and parafoveal and peripapillary vessel densities between patients with chiasmal compression and healthy controls.

	Patients (*n* = 57)	Healthy Controls (*n* = 42)	*p* value *
**RNFL thickness (μm)**	90.76 ± 10.68	99.96 ± 8.30	<0.001
**GCC thickness (μm)**	75.85 ± 8.37	83.77 ± 5.91	<0.001
**Vessel densities of SRCP (%)**	47.46 ± 2.03	48.67 ± 2.39	0.007
**Vessel densities of DRCP (%)**	48.77 ± 2.16	49.42 ± 2.18	0.141
**Vessel densities of RPC (%)**	56.91 ± 3.41	58.41 ± 2.55	0.020

*n* = numbers; RNFL = retinal nerve fiber layer; GCC = ganglion cell layer complex; SRCP = superficial retinal capillary plexus; DRCP = deep retinal capillary plexus; RPC = radial peripapillary capillary.* *p*-values by linear regression with adjustment for age and spherical equivalent.

**Table 3:** Association between postoperative visual field defects and demographic factors and ophthalmic parameters: univariate analysis.

Univariate Analysis	Estimate	Standard Error	*p* Value
**Gender**	0.002	0.998	0.998
**Age**	0.005	0.031	0.863
**Spherical equivalent**	−0.282	0.202	0.169
**Symptom duration**	−0.066	0.039	0.093
**Preoperative VF defects**	0.194	0.06	0.002
**pRNFL thickness**			
Average	0.059	0.037	0.122
Temporal	0.025	0.024	0.314
Inferior	0.034	0.025	0.167
Nasal	0.062	0.038	0.107
Superior	0.013	0.023	0.587
**GCC thickness**			
Average	0.066	0.05	0.192
Temporal	0.019	0.063	0.765
Inferior	0.056	0.049	0.258
Nasal	0.060	0.038	0.123
Superior	0.078	0.04	0.058
**SRCP**			
Average	0.468	0.19	0.017
Temporal	0.268	0.162	0.103
Inferior	0.232	0.126	0.072
Nasal	0.180	0.157	0.257
Superior	0.234	0.116	0.049
**DRCP**			
Average	0.284	0.185	0.131
Temporal	0.123	0.141	0.386
Inferior	0.121	0.111	0.281
Nasal	0.159	0.130	0.227
Superior	0.109	0.125	0.388
**RPC**			
Average	0.353	0.113	0.003
Temporal	0.142	0.082	0.088
Inferior	0.097	0.087	0.270
Nasal	0.129	0.064	0.051
Superior	0.138	0.063	0.032

VF = visual field; pRNFL = peripapillary retinal nerve fiber layer; GCC = ganglion cell layer complex; SRCP = superficial retinal capillary plexus; DRCP = deep retinal capillary plexus; RPC = radial peripapillary capillary.

**Table 4:** Association between postoperative visual field defects and demographic factors and ophthalmic parameters: multivariate analysis.

	Model 1^*^			Model 2^*^		
Variables	Estimate	Standard Error	*p* Value	Estimate	Standard Error	*p* Value
**Preoperative VF defects**	0.204	0.062	0.002	0.217	0.06	0.001
**Symptom duration**	NS	NS	NS	NS	NS	NS
**pRNFL thickness (nasal)**	NS	NS	NS	NS	NS	NS
**GCC thickness (superior)**	NS	NS	NS	NS	NS	NS
**RPC (average) (Model 1)**	NS	NS	NS	-	-	-
**RPC (superior) (Model 2)**	-	-	-	NS	NS	NS
**SRCP (average) (Model 1)**	0.444	0.191	0.025	-	-	-
**SRCP (superior) (Model 2)**	-	-	-	0.305	0.118	0.013

VF = visual field; pRNFL= peripapillary retinal nerve fiber layer; GCC = ganglion cell layer complex; SRCP = superficial retinal capillary plexus; RPC = radial peripapillary capillary; NS = not significant in multivariate analysis. * Final multivariable model only included variables with significant *p* value after conducting backward stepwise selection procedure.
